# Improving communication with patients with a hearing impairment

**Published:** 2013

**Authors:** Valerie E Newton, Seema Rupani Shah

**Affiliations:** Emeritus professor in audiological medicine: University of Manchester, Manchester, United Kingdom.; Audiological scientist, Nairobi, Kenya.

**Figure:**
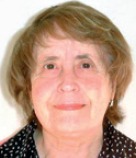
Valerie E Newton

**Figure:**
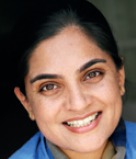
Seema Rupani Shah

The main barrier to communication for people who are hearing impaired is the lack of consideration by others. These patients can face prolonged or unnecessary illnesses due to inadequate communication with their health care providers.

However, by being prepared, and by preparing the patient, health workers can ensure good communication, thereby giving patients access to appropriate and effective health care.

Please note that patients with both visual and hearing impairments require even more consideration!

## Before the appointment

Patients with hearing impairments, with or without hearing aids, may communicate in a variety of ways with health personnel. Some patients speak and speech-read or lip-read, some use sign language or communicate by writing notes, and some bring someone with them to interpret. When advertising the eye clinic, or booking appointments, include information for patients on what to bring with them – such as their interpreter or their hearing aid.

## Reception and waiting areas

Waiting areas in clinics can be very noisy. Patients with severe or profound hearing loss will not hear shouted instructions or staff calling out their name. Those with moderate hearing loss can also have difficulty. The following general provisions may be helpful:

- In the waiting room, in addition to calling out the patient's name when it is his/her turn to be seen, use a number system or a sign (e.g. a board with the patient's name written on it).
- Write the most important information on clearly displayed signs.
- Put up a sign in your waiting room asking patients to inform you if they have a hearing impairment, and whether a sign language interpreter is available. Do not rely on being able to see a hearing aid – not all patients with hearing impairments wear these.
- If a patient with a hearing impairment calls in advance to make an appointment (or if someone else calls on his/her behalf), ask how the patient prefers to communicate and whether a sign language interpreter is needed.
- If you know a patient is hearing impaired, make sure the consultation takes place in a suitable setting (see next section).
- Keep a note on the patient's file with details of any communication requirements.

**Figure:**
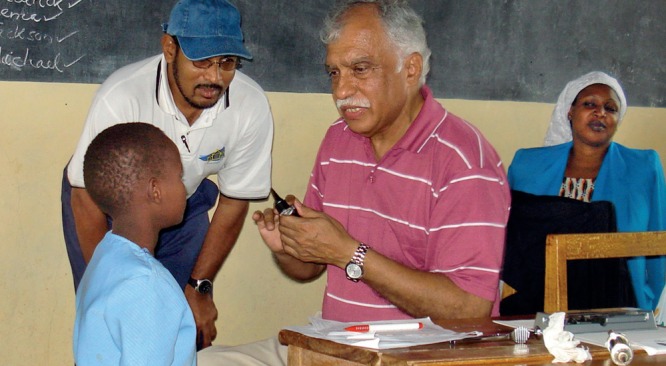
Examination of a deaf child in the presence of an interpreter. the doctor (right) is facing his young patient and has a pen and paper at hand. KENYA

## During an appointment

1. **The consultation room**
  - Give the patient privacy: they should be able to ask you to raise your voice without fear that others will hear about their medical history.
  - Minimise distractions. This is even more important if your patient is a child.
  - Reduce background noise.
  - Ensure the room is well lit, so that the patient can see your face or any written information they may be given.
  - Ask the patient to wear their hearing aids (if they have them and find them helpful) and sit closer to them than you would to another patient.
  - If possible, have a helper of the same gender as the patient in the room.
  - When adult patients are accompanied, always ask them before you start if they would prefer to be alone with health personnel in the consultation room. Do not wait until the questions become uncomfortable for the patient.
2. **Remember that your face is an essential communication tool**
  - Face the patient, not their interpreter or carer.
  - Remove any masks or face shields.
  - Do not have anything between your lips (cigarette, pen, etc.) or in your mouth (chewing gum, sweets, etc.) as this can distort lip movement when you are speaking. Avoid placing your hand or an object in front of your mouth when talking.
  - Have the light on your face rather than on the person you are talking to. This makes it easier for them to read facial expressions and to lip-read.
  - Support your speech with facial expression where you can, e.g. look happy if you are giving good news and sad if you are giving bad news.
  - When signing, hold your hands up at chest level to enable both your face and hands to be clearly seen.
  - Understand and use the local culture of gestures, expressions and accepted physical contact (or absence of it).
3. **Ensure that you speak effectively**
  - Speak normally, not too fast or too slowly. Certain sounds can be distorted or lost if speech is rushed or slowed down too much.
  - Use short, simple sentences.
  - Do not exaggerate your speech or lip movements.
  - Ask questions if you are not sure you understand what the patient is saying.
  - Patients tend to agree with their health are workers, sometimes without understanding what has been said to them. After every important point or message, ask the patient if he/she has understood you and, if necessary, ask him/her to repeat the message or instructions back to you (especially important if the patient is unaccompanied).
4. **Use other means of communication, e.g. writing and signing**
  - If the patient can sign, use an interpreter. If at all possible, learn the local sign language yourself. This can be fun and can be done with other colleagues.
  - Be prepared to write down any questions or answers, and give the person with a hearing impairment the opportunity to do the same if necessary.
  - Write down important information, e.g. instructions for taking medicines, to give to the patient. Have this information available in alternative formats (e.g. large print) for people with impaired vision or give the information to the person assisting them.
  - Use pictures and drawings to help the patient to understand you.

**PARTICIPATION**

**Figure:**
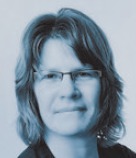
Diane Mulligan

Deputy Director, Advocacy and Alliances for Inclusive Development, CBM.

‘Nothing about us without us’ has been a slogan of the disability rights movement for decades. Participation is fundamentally about people with disabilities participating in decisions that relate to them so that actions affecting people with disabilities are not planned or performed without their input. This guiding principle highlights the need for people with disabilities to be brought into the process in such a way that they can directly influence decisions. This results in greater inclusion of people with disabilities and also brings with it lasting change.

Extensive involvement of people with disabilities will build skills and capacity. At the same time, people with and without disabilities working alongside each other can often foster changes in attitudes and understanding about the abilities, contributions, and aspirations of people with disabilities.

People with disabilities are often empowered and enabled by the confidence and skills that result from the fostering of genuine partnerships. These partnerships can include partnering with families, wider support networks, service providers, and community leaders, where appropriate. Working in partnership with disabled people's organisations (DPOs) is a very effective strategy (page 12).

